# De novo assembly of the Brown trout (*Salmo trutta* m. *fario*) brain and muscle transcriptome: transcript annotation, tissue differential expression profile and SNP discovery

**DOI:** 10.1186/s13104-020-05351-4

**Published:** 2020-11-02

**Authors:** J. Fibla, N. Oromi, M. Pascual-Pons, J. L. Royo, A. Palau, M. Fibla

**Affiliations:** 1 Institute of Biomedical Research of Lleida (IRBLleida), University of Lleida, Lleida, Spain; 2 Area of Biochemistry and Molecular Biology, School of Medicine, University of Malaga, Malaga, Spain; 3 Environment and Soil Sciences Department, ETSEA, University of Lleida, Lleida, Spain; 4 Animal Science Department, ETSEA, University of Lleida, Lleida, Catalonia Spain; 5 Complex Diseases Genetics, Departament de Ciències Mèdiques Bàsiques, Universitat de Lleida-IRBLLEIDA, Campus de Ciències de la Salut, Edifici Biomedicina I despatx b2.17, Av. Rovira Roure, 80, 25198 Lleida, Spain

**Keywords:** Rnaseq, De novo transcriptome, Brain & muscle transcriptome, SNP discovery, *Salmo trutta* m. *fario*

## Abstract

**Objectives:**

The Brown trout is a salmonid species with a high commercial value in Europe. Life history and spawning behaviour include resident (*Salmo trutta* m. *fario*) and migratory (*Salmo trutta* m. *trutta*) ecotypes. The main objective is to apply RNA-seq technology in order to obtain a reference transcriptome of two key tissues, brain and muscle, of the riverine trout *Salmo trutta* m. *fario*. Having a reference transcriptome of the resident form will complement genomic resources of salmonid species.

**Data description:**

We generate two cDNA libraries from pooled RNA samples, isolated from muscle and brain tissues of adult individuals of *Salmo trutta* m. *fario*, which were sequenced by Illumina technology. Raw reads were subjected to de-novo transcriptome assembly using Trinity, and coding regions were predicted by TransDecoder. A final set of 35,049 non-redundant ORF unigenes were annotated. Tissue differential expression analysis was evaluated by Cuffdiff. A False Discovery Rate (FDR) ≤ 0.01 was considered for significant differential expression, allowing to identify key differentially expressed unigenes. Finally, we have identified SNP variants that will be useful tools for population genomic studies.

## Objective

Brown trout (*Salmo trutta*) has been extensively studied by its commercial and biological importance. From the sixty-six species in this family, *S. trutta* is a species native to Europe with a wide distribution area that includes Atlantic and Mediterranean European basins, as well as northern Africa and western Asia basins [1, 2].The specie has been introduced in North and South America and Australia by its commercial exploitation for sport fishing, as well as farmed for food and game fish, extending their actual geographical distribution as discontinuous populations on all continents except Antarctica [3].

Life history traits of Brown trout populations include resident forms such as riverine (*S. trutta* m. *fario*) and migratory forms such as anadromous (*S. trutta* m. *trutta*) ecotype [4, 5]. Anadromous and non-anadromous forms coexist in the same river being apparently genetically indistinguishable [6, 7]. An extended literature on Brown trout research has been produced that includes physiological, ecological and genetic aspects [8–10]. As a contribution to this global effort, here we provide a comprehensive transcriptome data set derived from brain and muscle tissues of *Salmo trutta* m. *fario* ecotype by using RNA-seq technology. We also evaluated differential transcript expression among these two tissues identifying key differentially expressed unigenes. Finally, we applied an in-silico pipeline that allow us to discover SNP variants useful for population genomic studies. The generated data could provide new valuable genomic resources for population genetic and genomic studies that can help to answer opened questions about the live history traits of riverine *S. trutta* m. *fario* as well as differences among *S. trutta* ecotypes.

## Data description

*Salmo trutta* m. *fario*. brain and muscle tissues were collected from 25 wild type individuals (15 females) captured at the Falmisell river (Lleida, Catalonia). RNA pools from brain (10.2 µg) and muscle (11.4 µg) tissues were obtained with equimolar concentration from each subject. The TruSeq™ RNA sample Prep Kit (Illumina, Madrid, Spain) was used to build cDNA libraries according to manufacturer instructions (Table 1, Data file 1). FASTQ sequence reads were assembled using Trinity [11] run on the paired end sequences with the fixed default k-mer size of 25 and minimum contig length of 200. Descriptive statistics of assembly and sequencing is found at Table 1 (Data file 2 and Data file 3). Among the 144,984 contigs predicted by Trinity (Table 1, Data file 4 and Data file 8), we identify protein coding regions using TransDecoder package [11]. We retained the longest ORF predicted for each contig sequence with a minimum of 100 amino acids long. Transcript redundancy was further reduced by CD-hit [12], obtaining a final set of 35,189 non-redundant ORF unigenes as best cluster representatives (Table 1, Data file 5). Size distribution for clustered ORF unigenes is presented in Table 1 (Data file 3). This final set was characterized by homology search to nucleotide and protein databases (Table 1, Data file 10 and Data file 11). Taxonomic representation showed the top hits for a large fraction of unigenes (≈88%) to *Neopterigii* taxon, with 66% of unigenes assigned to family *Salmonidae* (*Salvelius sp*. (1%), *Onchorrinchus sp*. (14%) and *Salmo sp.* (51%) (Table 1, Data file 12). A total of 4337 protein motif were assigned to 23,616 ORF unigenes, being the RNA recognition motif (6.4%), Immunoglobulin domain (4.8%), Tetratricopeptide repeat (4.8%) and Protein kinase domain (3.4%) the most prevalent (Table 1, Data file 13).

**Table 1:** Overview of data files/data sets

Label	Name of data file/data set	File types (file extension)	Data repository and identifier (DOI or accession number)
Data file 1	Methodology description	Document file (.docx)	*Figshare* https://doi.org/10.6084/m9.figshare.12902474.v1
Data file 2	Descriptive statistics of assembly-sequencing	Document file (.docx)	*Figshare* https://doi.org/10.6084/m9.figshare.12902474.v1
Data file 3	FigS1 Size_distribution	Image file (.jpg)	*Figshare* https://doi.org/10.6084/m9.figshare.12902405.v2
Data file 4	FigS2 GeneOntology	Image file (.jpg)	*Figshare* https://doi.org/10.6084/m9.figshare.12902405.v2
Data file 5	FigS3 Differential_expression	Image file (.jpg)	*Figshare* https://doi.org/10.6084/m9.figshare.12902405.v2
Data file 6	Raw RNA‐seq. Reads Brain tissue	Fastq files (.fastq)	*NCBI Sequence Read Archive* https://identifiers.org/insdc.sra:SRP151838
Data file 7	Raw RNA‐seq. Reads Muscle tissue	Fastq files (.fastq)	*NCBI Sequence Read Archive* https://identifiers.org/insdc.sra:SRP151838
Data file 8	Trinity144	Fasta file (.fasta)	*Figshare* https://doi.org/10.6084/m9.figshare.7326464
Data file 9	Predicted non-redundant Open Reading Frames (ORFs)	Fasta file (.fasta)	*NCBI GenBank* https://identifiers.org/ncbi/insdc:GHGR00000000.1
Data file 10	Megablast hit aligment of non-redundant ORF unigenes to reference nucleotide databases	Spreadsheet (.xlsx)	*Figshare* https://doi.org/10.6084/m9.figshare.7712708.v4
Data file 11	Blastx homology search of non-redundant ORF unigenes to reference protein databases	Spreadsheet (.xlsx)	*Figshare* https://doi.org/10.6084/m9.figshare.7712708.v4
Data file 12	Krona_pie_chart_on_Non_redundant_ORF_to_NCBI_nt_and_rnaREF_seq_2018__HTML_html	HTML file (.html)	*Figshare* https://doi.org/10.6084/m9.figshare.7712708.v4
Data file 13	Protein family (Pfam) assignation to non-redundant ORF unigenes	Spreadsheet (.xlsx)	*Figshare* https://doi.org/10.6084/m9.figshare.12905777.v2
Data file 14	GOslim annotation of non-redundant ORF unigene sequences	Spreadsheet (.xlsx)	*Figshare* https://doi.org/10.6084/m9.figshare.12905777.v2
Data file 15	KEGG pathway annotation of non-redundant ORF unigene sequences	Spreadsheet (.xlsx)	*Figshare* https://doi.org/10.6084/m9.figshare.12905777.v2
Data file 16	Raw_Cufflinks_Brain_transcript_expression	Cufflinks output file (.txt)	*Figshare* https://doi.org/10.6084/m9.figshare.12905747.v1
Data file 17	Raw_Cufflinks_Muscle_transcript_expression	Cufflinks output file (.txt)	*Figshare* https://doi.org/10.6084/m9.figshare.12905747.v1
Data file 18	Raw_Cuffdiff_Brain_Muscle_transcript_differential_expression_testing	Cuffdiff output file (.txt)	*Figshare* https://doi.org/10.6084/m9.figshare.12905747.v1
Data file 19	Differentialy expressed non-redundant ORF unigenes at FDR_0.01	Spreadsheet (.xlsx)	*Figshare* https://doi.org/10.6084/m9.figshare.12905747.v1
Data file 20	Salmo trutta m. Fario—mapped SNP_to_ORF	Varian Call Format file (.vcf)	*Figshare* https://doi.org/10.6084/m9.figshare.12905831.v1
Data file 21	SNP context sequence	Spreadsheet (.xlsx)	*Figshare* https://doi.org/10.6084/m9.figshare.12905831.v1

Similarity search by Blast2GO renders a total of 28,132 (80%) unigenes with GO annotation. GO term were then simplified using a generic GOSlim vocabulary [13] (Table 1, Data file 14). The ten top GO terms among the Cellular Component (18,071, 64%), Molecular Function (20,691, 74%) and Biological Process (23,954, 85%) ontology at level 2 are shown in Table 1 (Data file 4). Mapping unigenes to the reference canonical pathways in the KEGG database, yields a total of 13,957 (39.8%) ORF unigenes assigned to 3421 KEGG terms (KO) defining a total of 386 pathways (Table 1, Data file 15).

Tissue specific transcriptome expression analysis was performed by normalization of raw reads (FPKM, fragments per kilobase of exon per million fragments) obtained from both tissues (Table 1, Data file 16 and Data file 17). Analysis reveals 1172 ORF unigenes expressed only in muscle, 8595 expressed only in brain and 12,072 expressed in both tissues (Table 1, Data file 5, FigS3). Differentially expressed unigenes at FDR < 0.01 and best homologous sequences are shown at Table 1 (Data file 18 and Data file 19).

Finally, we have identified 73,237 putative SNPs (Table 1, Data file 20) and extracted 150 bp sequence context to each SNP as a source for the design of PCR primers useful for genotyping protocols (Table 1, Data file 21).

## Limitations

The use of pooled RNA samples does not allow us to detect sex or individual specific transcript expression profiles as well as limit our capability to detect transcripts expressed at low level in a specific individual. In addition, pooled samples avoid us to resolve SNP frequency distribution, being this parameter indirectly estimated according to the observed SNP sequence coverage in the pooled sample.

## Data Availability

The data described in this Data note can be freely and openly available on *Figshare* (https://doi.org/10.6084/m9.figshare.12902474.v1; https://doi.org/10.6084/m9.figshare.12902405.v2; https://doi.org/10.6084/m9.figshare.7326464.v1; https://doi.org/10.6084/m9.figshare.7712708.v4; https://doi.org/10.6084/m9.figshare.12905777.v2; https://doi.org/10.6084/m9.figshare.12905747.v1; https://doi.org/10.6084/m9.figshare.12905831.v1). Assembly of non-redundant ORF unigene sequences are available from NCBI transcriptome shotgun assembly (TSA) database (https://identifiers.org/ncbi/insdc:GHGR00000000.1). Raw sequence reads are available from the NCBI sequence read archive (SRA) database (https://identifiers.org/insdc.sra:SRP151838). Please see Table 1 and references list [14–22] for details and links to the data.

## Abbreviations

BAM: Binary Sequence Alignment/Map
BLAST: Basic local alignment search tool, bp: base pair
CDS: Coding sequence
FPKM: Fragments Per Kilobase of exon model per Million mapped reads
GO: Gene Ontology
KEGG: Kyoto Encyclopaedia of Genes and Genomes
ORF: Open reading frame
Pfam: Protein families database
FDR: False Discovery Rate
SAMtools: Sequence Alignment/Map tools
SNP: Single nucleotide polymorphism
